# Serological Evidence for Multiple Strains of Canine Norovirus in the UK Dog Population

**DOI:** 10.1371/journal.pone.0081596

**Published:** 2013-12-05

**Authors:** Sarah Caddy, Edward Emmott, Laila El-Attar, Judy Mitchell, Alexis de Rougemont, Joe Brownlie, Ian Goodfellow

**Affiliations:** 1 Division of Virology, Department of Pathology, University of Cambridge, Addenbrookes Hospital, Hills Road, United Kingdom; 2 Section of Virology, Faculty of Medicine, Imperial College London, St. Mary's Campus, London, United Kingdom; 3 Department of Pathology and Pathogen Biology, The Royal Veterinary College, Hatfield, Hertfordshire, United Kingdom; 4 National Reference Center for Enteric Viruses, Laboratory of Virology, University Hospital of Dijon, University of Bourgogne, Dijon, France; University of Liverpool, United Kingdom

## Abstract

Noroviruses are associated with intestinal disease in humans, cows, pigs, mice, and, more recently, dogs. In 2007, the first canine norovirus (CNV) was identified and characterized in Italy. Subsequent studies have identified CNV in stools of dogs from Portugal, Greece, and the United States. To investigate the prevalence of CNV in the UK dog population, 228 canine stool samples were screened for CNV by qPCR, and 396 serum samples were screened for anti-CNV antibodies. qPCR of RNA extracted from canine stool samples did not reveal any CNV-positive samples, based on samples collected from diarrhoeic and control dogs in 2012–2013. CNV virus-like particles to three different CNV strains were produced using recombinant baculoviruses and a seroprevalence screen undertaken. Anti-CNV antibodies were identified at significant levels in canine serum; 38.1% of samples collected between 1999–2001 and 60.1% of samples collected in 2012–2013 were seropositive. The increase in seroprevalence over time (*p*<0.001) suggests that the CNV strains screened for are becoming more widespread. Variation in seroprevalence to different CNV strains was also identified. Two-thirds of the dogs were seropositive to a single strain, whereas the remaining third were seropositive to two or three of the strains analysed. This study has provided the first evidence that CNV is present in the UK, with seroprevalence identified to multiple circulating strains. This warrants further study and increased awareness of this recently discovered canine virus.

## Introduction

Noroviruses are members of the RNA virus family *Caliciviridae*, and are a major cause of human infectious gastroenteritis worldwide. An estimated three million people each year in the UK suffer from ‘winter vomiting disease’ caused by human norovirus [1]. Infection causes the classic symptoms of vomiting, diarrhea and malaise, and outbreaks are common in closed or semi-closed communities such as in hospitals, care homes, schools and cruise ships. In addition to the major burden of noroviruses on human health, noroviruses have also been found associated with intestinal disease in cows [2], pigs [3], mice [4], a lion [5], cats [6] and dogs [7]. The first canine norovirus (CNV) was reported from a single dog with enteritis in Italy in 2007. Subsequent studies have identified CNV in stools of dogs from Portugal [8], [9], Greece [10] and the US [11]. To date there have been no reports of CNV present in the UK.

Significant sequence variation has been found in different CNV strains identified to date. Noroviruses are assigned to six genogroups based on complete capsid sequences, with strains of norovirus assigned to the same genogroup if they share 55–85% amino acid identity [12]. Human noroviruses are grouped together in genogroups I, II and IV whereas CNV strains have been assigned to genogroups IV and VI. Human and canine noroviruses in genogroup IV share <85% amino acid identity, thus are separated into different genotypes, IV.1 and IV.2 respectively. Genetic recombination is believed to occur between different norovirus strains, which may explain the significant heterogeneity between the CNV strains identified [13].

The prevalence of CNV in dogs with clinical signs of gastroenteritis across Europe has been estimated to be between 2.1% (Italy [13]) and 40% (Portugal [8]). A study in the US identified CNV at a prevalence of 11% in canine diarrhoea samples [11]. CNV has also been detected in healthy dogs [8], and the association between infection and clinical signs is yet to be formally established. CNV has been identified in dogs also infected with other enteric viral pathogens such as canine parvovirus (CPV) and canine enteric coronavirus (CECoV), thus elucidating the role of CNV infection in disease is difficult.

Serological prevalence of CNV in countries where the virus has been detected has not been reported. A preliminary serological survey in Italy suggested less than 5% dogs were seropositive to a genogroup IV.2 lion norovirus (strain Pistoia/387/06/ITA) but the sample size was small [14]. As with human norovirus, CNV has yet to be cultivated in cell culture thus obtaining sufficient quantities of virus for serological screening is not possible. However, the major capsid protein of noroviruses (VP1) has been shown to spontaneously assemble into virus-like particles (VLPs) when recombinant VP1 is expressed in an appropriate system [15]. Production of lion and canine norovirus VLPs has previously been achieved and proven to be an efficient way of generating antigen for serological analysis [14], [16].

The aim of this study was to elucidate the importance of CNV in the UK dog population and evaluate any changes over time. No prior information on CNV prevalence in the UK has been described, so in order to address this our study has sought to determine the prevalence of CNV RNA in canine fecal samples, in conjunction with the prevalence of anti-CNV antibodies in different populations of dogs.

## Methods

### Ethics Statement

No ethical approval was required for this study as all clinical samples collected were either animal waste products, surplus to diagnostic requirements, or derived from a previously published and ethically approved study [17].

### Samples

Stool samples were collected from dogs admitted to four participating veterinary clinics in geographically distinct areas across the UK. With owner consent, dogs were recruited to the study if they passed stools whilst hospitalized. Stools were collected by veterinary personnel, then stored at −20°C until and during transportation to the laboratory, whereafter they were stored at −80°C prior to nucleic acid extraction. Stool samples were also collected from healthy dogs owned by veterinary staff at each clinic, as well as from dogs at participating boarding kennels. Basic case data was recorded for each dog from which a stool sample was collected, including age, breed, sex, reason for admission, and any recent history of enteric disease.

Serum samples were obtained from two separate dog populations. Samples from 1999–2001 were collected from a rehoming kennel as part of an existing study [17]. Serum samples from 2012–2013 were obtained from the diagnostic service of the Royal Veterinary College, and from the UK Pet Blood Bank. These sera were collected from pet dogs that were either blood donors, or veterinary patients from which blood was collected for biochemical analysis for various reasons.

### Nucleic Acid Extraction and qRT-PCR

Stools were diluted 10% w/v in phosphate-buffered saline, pH 7.2, and solids were removed by centrifugation at 8000×g for 5 min. Viral nucleic acid was extracted from 140 µl of each clarified stool suspension by the GenElute™ Mammalian Total RNA Miniprep Kit (Sigma Aldrich) according to the manufacturers’ instructions.

An internal extraction control was added to each sample during nucleic acid extraction to verify removal of PCR inhibitors and enable precise quantification of viral nucleic acid. A fixed amount of Equine Arteritis Virus (EAV) RNA was added with the lysis buffer to each sample to obtain an EAV concentration of approximately 1×10^8^ copies per ml of faecal suspension.

qRT-PCR was selected as the screening method as its high sensitivity allows for the detection of lower viral copy numbers than conventional PCR. All 6 CNV sequences listed in Genbank in August 2012 (table 1) were used to design a CNV specific primer-probe (PrimerDesign Ltd, table 2). The target sequence is the highly conserved region of the RdRp (NS7). A positive control amplicon was used to generate a standard curve. As few as 100 copies of the CNV amplicon were reliably detected in a reaction volume of 20 µl. In addition to CNV, samples were also screened for two other canine enteric viruses known to be circulating in the UK. Primer-probes used for canine parvovirus (CPV) and canine enteric coronavirus (CECoV) are listed in table 2, as well as the primer-probe sequence used to detect the internal extraction control.

**Table 1:** Overview of CNV strains used to design primer-probes and produce virus-like particles (VLP).

CNV strain	Used in primer-probe design	Used in VLP production (abbreviation)	GenBank Accession Number
GIV.2/Bari/170/07-4/ITA	Yes	Yes (170)	EU224456.1
GVI.1/Bari/91/2007/ITA	Yes	No	FJ875027.1
FD210/2007/Ita	Yes	No	JF939046
FD53/2007/Ita	Yes	No	JF930689
C33/Viseu/2007/PRT	Yes	Yes (C33)	GQ443611.1
Thessaloniki/30/2008/GRC	Yes	No	GU354246.1
GVI.1/HKU_Ca035F/2007/HKG	No	Yes (HK)	FJ692501.1

Six CNV sequences were used to design the primer-probe set used for qPCR detection. The strain GVI.1/HKU_Ca035F/2007/HKG had not been reported at time of design. Three of the most diverse CNV sequences were selected for VLP production.

**Table 2:** Primers and probe sequences used in qPCR screen of canine stool samples for enteric viruses.

Virus	Primer/Probe	Ref
Canine norovirus	F: GCTGGATGCGGTTCTCTGACR: TCATTAGACGCCATCTTCATTCACProbe: FAM-AGCGAGATTGCGATCTCCCTCCCACAT-BHQ	This report
Canine parvovirus	F: AAACAGGAATTAACTATACTAATATATTTAR: AAATTTGACCATTTGGATAAACTProbe: VIC-TGGTCCTTTAACTGCATTAAATAATGTACC-BHQ	[32]
Canine entericcoronavirus	F: TTGATCGTTTTTATAACGGTTCTACAAR: AATGGGCCATAATAGCCACATAATProbe: Cy5-ACCTCAATTTAGCTGGTTCGTGTATGGCATT -BHQ	[32]
Equine arteritis virus(internal control)	F: CATCTCTTGCTTTGCTCCTTAGR: AGCCGCACCTTCACATTGProbe: Cy5.5-CGCTGTCAGAACAACATTATTGCCCAC-BHQ2	[33]

Four primer-probe sets were used in the viral nucleic acid survey. CPV, CECoV and EAV primer-probe sets have previously been published whereas the CNV primer-probe set was designed for this study.

A 1-step qRT-PCR protocol was used to improve ease and efficiency of sample handling. 2 µl of extracted RNA was added to 2× Precision OneStep qRT-PCR MasterMix (PrimerDesign Ltd), 6 pMol/µl primers, and 3 pMol/µl probe. The thermal cycle protocol used with a ViiA7 qPCR machine (AB Applied Biosystems), was as follows: 55°C for 30 mins, inactivation of reverse transcriptase at 95°C for 5 mins, and then 40 cycles consisting of denaturation at 95°C for 15 s, then annealing and elongation at 60°C for 1 min.

### Virus-like Particle (VLP) Production

Three different CNV strains with maximum sequence difference were selected for VLP production. The sequences of the three CNV-VP1 genes were obtained from GenBank (table 1) and restriction enzymes sites designed (5′ NotI for all strains, 3′ BbsI for CNV strains 170, C33 and 3′ BsaI for CNV HK) to enable later ligation into the baculovirus transfer vector pTriex1.1. Sequences were synthesized by BioBasic Inc. in the vector pUC57. CNV-VP1 sequences were digested from pUC57 and re-ligated into pTriex1.1 that had been digested with NcoI and NotI (NEB). The correct sequence for all three CNV-VP1 inserts was confirmed by sequencing.

Recombinant baculoviruses were generated using the *flash* BAC baculovirus expression system as per the manufacturers instructions (Oxford Expression Technologies). Stock viruses were generated and titrated in Sf9 cells and stored in the dark at 4°C. Protein expression was performed in Hi5 insect cells (Invitrogen). Briefly, 1×10^7^ Hi5 insect cells were seeded into 10×T150 flasks then infected with recombinant baculovirus at a multiplicity of infection of 5 pfu/cell. Infections were allowed to proceed for 6 days prior to protein harvest and VLP purification. VLP purification was performed essential as described [18]. VLP was released from infected Hi5 cells by freeze-thaw, followed by clarification to remove cellular debris (6000×g, 30 minutes) then baculovirus removal (14,000×g for 30 mins). VLPs were partially purified through a 30% w/v sucrose cushion in TNC buffer (50 mM Tris HCl pH 7.4, 150 mM NaCl, 10 mM CaCl_2_) containing the protease inhibitor leupeptin for 150,000×g for 2 hrs. The pelleted VLP was resuspended in TNC and further purified by isopynic centrifugation in caesium chloride (150,000×g, 18 hrs). The resultant VLP bands were collected by puncture and the solution containing VLPs was dialysed against PBS prior to quantification by BCA protein assay (Thermo Scientific) and storage at −80°C.

### ELISA Procedure

Ninety-six-well polystyrene microtiter plates (Nunc maxisorb, Fisher Scientific) were coated overnight at 4°C with 75 ng of pooled CNV VLPs consisting of 25 ng of each strain; 170, C33 and HK in 0.05 M carbonate/bicarbonate buffer (pH 9.6). Plates were washed three times with 0.05% Tween 20 in phosphate buffered saline (PBS-T) before blocking in 5% skimmed milk-PBS-T for 1 h at 37°C and then three PBS-T washes. Plates were then incubated for 3 h at 37°C with 1∶50 dilution of each serum sample in duplicate in 5% skimmed milk-PBS-T. Pooled human sera (Sigma Aldrich), diluted 1∶400, and 100 ng pooled GII human norovirus VLPs were used as a positive control until a canine positive control was identified. After three washes with PBS-T, 50 µl of horseradish peroxidase (HRP)-conjugated anti-dog IgG antibody (Sigma Aldrich) diluted 1∶5000 in 5% milk PBS–T, was added to each well and incubated at 37°C for 1 h. The plates were washed four times with PBS-T and bound antibody detected with 50 µl tetramethylbenidine (TMB, Sigma Aldrich) followed by incubation at room temperature for 10 min. The reaction was stopped with 1 N H_2_SO_4_ and the optical density (OD) was read at 450 nm (Spectromax M2 plate reader, Molecular Devices).

To eliminate the possibility that non-specific components of the VLP preparation were identified by the canine sera, an antigenically distinct vesivirus 2117 VLP was included in the assay. The OD450 of a selection of serum samples incubated on either carbonate/bicarbonate buffer coated wells or vesivirus 2117 coated wells was highly comparable. This confirmed that no non-specific reactivity relating to the VLP preparation was occurring. The background signal for each sample was hence determined by measuring the OD450 of serum samples incubated with carbonate/bicarbonate buffer alone. Background signal was then subtracted from the OD450 of VLP coated wells to generate the corrected OD450 value. A threshold value was established as the mean of the OD450 of all buffer coated cells plus 3 standard deviations. A serum sample was considered positive when the corrected OD450 was higher than the threshold. Any serum samples showing a positive response to pooled CNV VLPs were subjected to further testing with individual CNV VLPs. Plates were coated with 25 ng of individual VLPs in carbonate/bicarbonate buffer and the protocol then repeated as above.

Evaluation of serological cross reactivity between different norovirus strains was achieved using VLP competition assays. Plates were coated with 25 ng/well of VLP overnight at 4°C. CNV positive canine sera was incubated with a range of concentrations of each of the either human norovirus VLPs, or individual CNV VLPs (0.5, 1, 2 and 4 µg/ml) for 1 h at 37°C. Vesivirus 2117 VLP was incubated with the canine sera as a negative control. After the incubation period, 50 µl of each serum-VLP combination was added to the previously VLP coated plates. The remainder of the ELISA protocol was followed as detailed above.

### SDS-PAGE and Western Blot Analysis

VLPs were heated to approximately 100°C for 5 min in the presence of SDS loading buffer and electrophoresed on 12.5% SDS-polyacrylamide gels. For Coomassie blue staining, the gels were incubated with Coomassie Blue for 1 h at room temperature prior to de-staining. For western blotting, proteins were transferred from SDS-polyacrylamide gels to polyvinylidene difluoride membranes. The membranes were blocked for 1 h at room temperature with 5% milk in PBS–Tween 20 (0.1%) and then incubated overnight at 4°C with serum samples diluted 1∶1000. The excess antibody was washed three times in PBS–T and incubated for 1 h with anti-canine IgG secondary antibody conjugated to horseradish peroxidase (Sigma Aldrich). After washing away excess secondary antibody, the bands were detected using enhanced chemiluminescence reagent (GE Healthcare).

## Results

### Samples Collected

Stool samples and clinical data were collected from 111 dogs admitted to veterinary clinics distributed across the UK between August 2012 and March 2013. The mean age of the dogs was 5.2 years (standard deviation 4.2 years), with 42 different breeds represented. Diarrhoea and/or vomiting lasting more than 24 hrs prior to admission, was reported in 52% of these cases. The gastrointestinal clinical signs in a proportion of these cases were unlikely to be attributable to primary gastroenteritis (e.g. pyometra, foreign body ingestion), thus overall it was found that 43% of dogs enrolled in the study were suffering from gastroenteritis, infectious or otherwise. Control samples were collected from 117 healthy dogs (mean age 5.6 years, standard deviation 3.6 years) from boarding kennels or belonging to veterinary staff.

### Viral RNA Survey

Nucleic acid extraction and qPCR were successfully performed on 228 stool samples as determined by constant C_t_ values from the internal extraction control RNA. In addition to CNV, samples were systematically tested for the presence of canine parvovirus (CPV) and canine enteric coronavirus (CECoV). Table 3 summarises the results obtained. Enteric viruses, either CPV or CECoV, were detected at high titre (>10^7^ copies/ml stool) in 17.0% (8/47) of dogs admitted with primary gastroenteritis. No viruses were detected at significant titres in patients without gastroenteritis or in the healthy control dogs. No samples were positive for CNV viral RNA using the primer set described in Table 2. This indicates that the overall prevalence of CNV in this population at the time of sample collection was <1.7% (Wilson binomial approximation, confidence interval 95%).

**Table 3:** Viruses detected in dogs with and without gastroenteritis.

Sample group (size)	Canine norovirus	Canine parvovirus	Canine enteric coronavirus
Patients with gastroenteritis (47)	0	7	1
Patients without gastroenteritis (64)	0	0	0
Healthy controls (117)	0	0	0

qPCR was performed on nucleic acid extracted from stools of 228 dogs. Of these dogs, 47 were admitted to veterinary clinics with primary gastroenteritis, 64 dogs were admitted to veterinary clinics with other clinical disease, and 117 dogs were healthy animals. Primer probes specific for canine norovirus (CNV), canine parvovirus (CPV) and canine enteric coronavirus (CECoV) were used to assay for the enteric viruses.

### Production and Purification of VLPs from VP1 of Three CNV Strains

**Figure 1:**
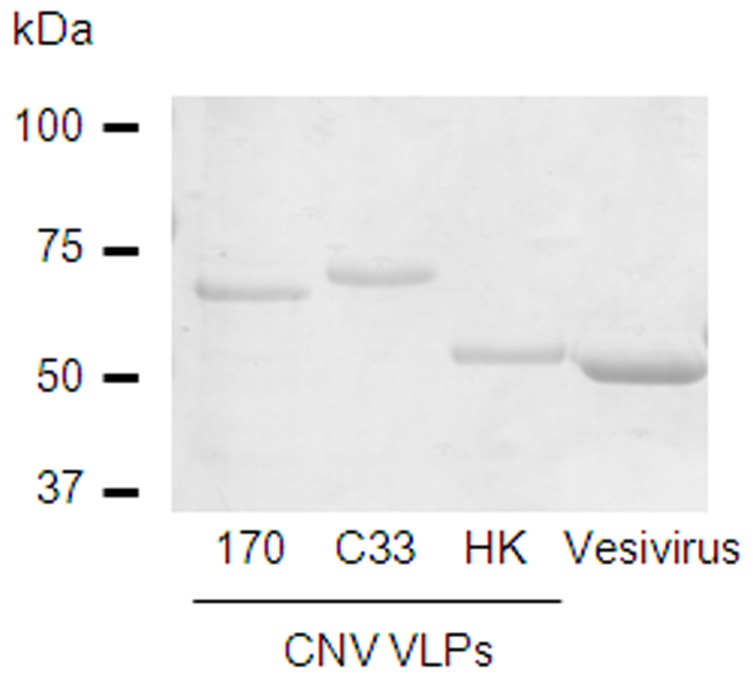
SDS-PAGE analysis of purified calicivirus VLPs. VLPs from three CNV strains and an unrelated calicivirus, vesivirus 2117, were analysed by SDS-PAGE. The molecular weights of VP1 of CNV isolates C33 and 170 are larger than that of the third isolate HK. This is attributed to the length of C33 and 170 VP1 sequences being 52 and 50 amino acids respectively longer than HK VP1.

Recombinant baculoviruses expressing the VP1 proteins from three distinct CNV isolates were generated and used in an insect cell expression system to produce CNV VLPs. Following purification by isopycnic centrifugation on a CsCl gradient, VLPs were readily visualized and sedimented with a density of 1.32 g/ml. Purified VLPs were quantified by spectrophotometry, and examined by SDS-PAGE and staining with Coomassie blue (figure 1). This showed a single major protein with apparent molecular weight of 63 kDa for CNV strains 170 and C33, and single major protein with apparent approximate molecular weight of 57 kDa for strain HK. This difference in molecular weight was expected based on VP1 sequence length. VLPs of an unrelated calicivirus, vesivirus 2117, were included as a control.

### Serological Survey

A total of 396 canine sera were tested by ELISA for antibodies against CNV, 223 from a 1999–2001 cohort, and 173 from a 2012–2013 cohort. As part of an initial screen, CNV VLPs to three strains (177, C33 and HK) were combined and a 1∶50 dilution of canine sera examined for their reactivity to CNV. Overall 189 were found to be seropositive to CNV. In the 1999–2001 cohort, 85 dogs (38.1%) were seropositive and in the 2013/2013 cohort, 104 (60.1%) dogs were seropositive. The increase in seroprevalence between the two cohorts was statistically significant (Z-test, *p*<0.001).

**Figure 2:**
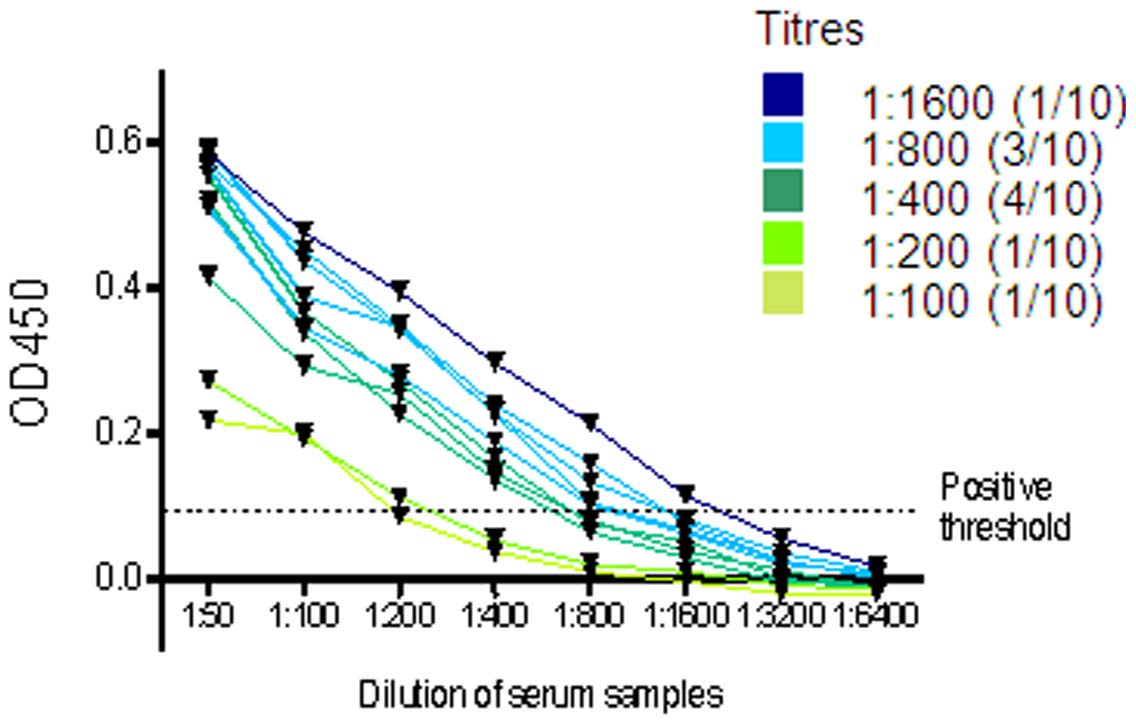
Serological titres to CNV. Ten dogs seropositive to CNV from both the 1999–2001 and the 2012–2013 cohorts were randomly selected to determine anti-CNV antibody titre. Corrected OD450 values are plotted, calculated by subtracting the OD450 value of buffer-only coated wells. The positive threshold was calculated from the mean plus three times the standard deviation of the OD450 reading of buffer-only.

To determine the serological titres of seropositive dogs, ten seropositive samples were randomly selected from both the 1999–2001 cohort and the 2012–2013 cohort. Sera was serially diluted two-fold from a starting dilution of 1∶50, and added to pooled CNV VLPs (25 ng each strain per well) coated onto 96-well plates. A range of titres were identified (figure 2), varying from 1∶1600 (1 dog) to 1∶100. The most prevalent titre in this preliminary screen was 1∶400.

**Figure 3:**
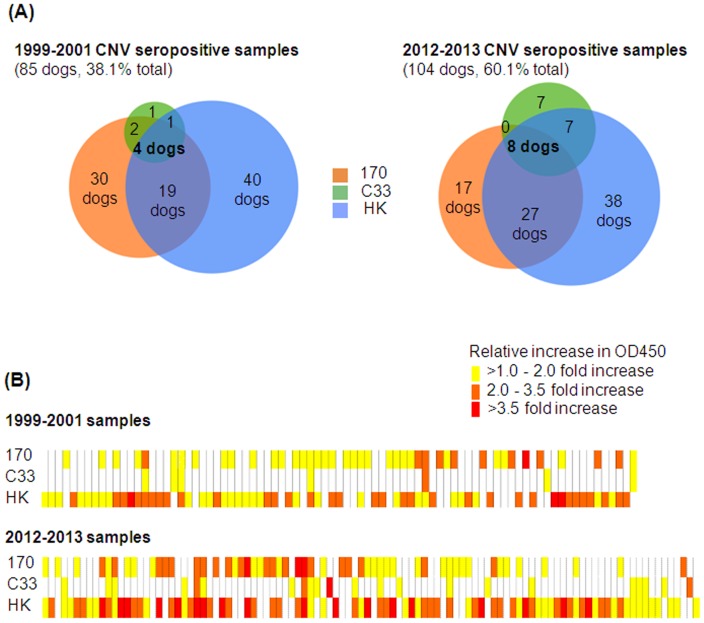
Seropositive samples by ELISA to three different strains of CNV. Canine serum samples that were seropositive to pooled CNV VLPs were screened against individual CNV VLP strains; 170, C33 and HK. (A) Venn diagrams represent seroprevalence of each CNV strain in the 1999–2001 and the 2012–2013 cohorts. The number of dogs seropositive to one strain alone or combinations of strains are represented as percentages. (B) The relative OD450 value to each CNV strains of each dog are represented by heatmaps. Each column represents a single dog. Positive threshold value was established from the mean OD450 of coating buffer alone plus three standard deviations. Relative increase in OD450 values above the positive threshold were calculated to enable fair comparison between experiments. A relative increase of <1 indicates a seronegative sample, represented by a white box. The degree of relative increase for samples is represented by increasing darkness of the corresponding box.

All 189 serum samples identified as positive by the initial ELISA screen against pooled CNV VLPs, were then tested against individual CNV VLPs. The results show significant variation between the seroprevalence of different strains (figure 3). CNV strain HK predominated in both cohorts of dogs, whereas strain C33 showed the lowest seroprevalence in both groups. These data also indicated that a proportion of dogs have seroconverted to more than one of the CNV strains used in this study. In the 1999–2001 cohort 30.6% dogs were seropositive to 2 or 3 strains, a percentage that increased to 40.4% in the 2012–2013 cohort.

### Evaluation of CNV Antibody Cross Reactivity

**Figure 4:**
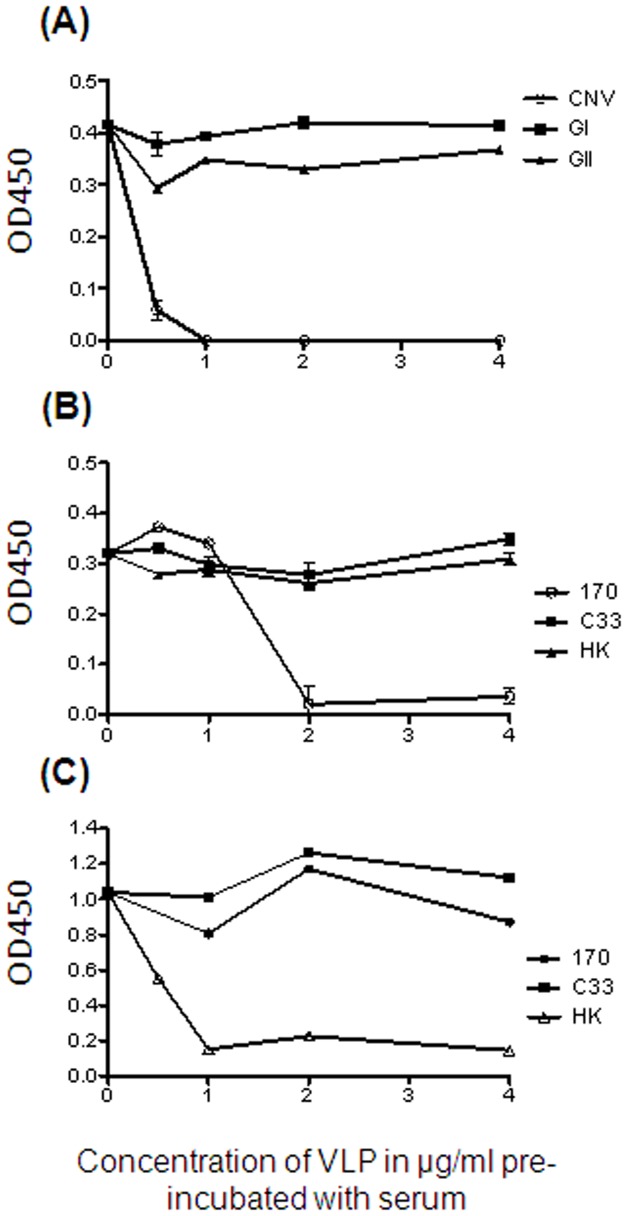
Evaluation of cross-reactivity between antibodies against human and canine noroviruses, and between different CNV strains. Canine serum was pre-incubated with serial diutions of either pooled human norovirus VLPs from genogroups I and II (GI/GII) or pooled CNV VLPs. The ability to detect pooled CNV was analysed using ELISA (A). Canine serum was then pre-incubated with serial dilutions of each of the CNV strains VLPs separately. ELISAs were again used to analyse the ability to detect CNV strain 170 (B) and strain HK (C). No C33 seropositive sample of adequate titre was available for the blocking assay.

To determine the strain specificity of the anti-CNV reactivity identified in positive samples, a series of blocking assays were performed on two selected samples. Firstly it was shown that pre-incubating a serum sample positive to pooled CNV VLPs with human norovirus VLPs from genogroup I and II, did not diminish signal to CNV (figure 4A). This demonstrates that apparent reactivity to CNV is not a consequence of cross-reactivity to human norovirus strains. To investigate the antigenic relationship between different CNV strains, blocking assays were next performed using individual CNV strains. When a CNV seropositive sample was pre-incubated with CNV strain 170 VLP, subsequent detection in ELISA of strain 170 VLP coated on a plate was reduced. Pre-incubation with VLPs of a different CNV strain did not diminish signal. A similar result was shown when serum was pre-incubated with CNV strain HK VLPs; detection of HK VLPs by ELISA was reduced (figure 4B and 4C). No serum that contained anti-CNV strain C33 antibodies was available in sufficient quantities for the blocking assay, though the results from pre-incubation with C33 VLPs were sufficient to indicate that cross reactivity to strain 170 or HK was not occurring. This data overall demonstrates that anti-CNV antibodies against the three strains used in this study are strain specific, suggesting that CNV exists as distinct serotypes.

**Figure 5:**
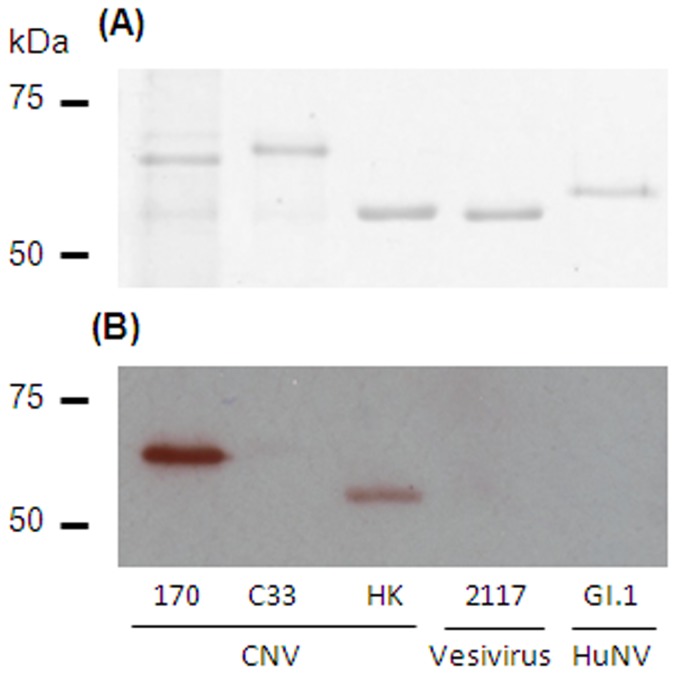
Western blotting of purified VLPs using seropositive canine serum. Five different VLPs were separated by SDS-PAGE (2 µg each). Gel A was stained with Coomassie blue to identify purified VLP protein at the expected molecular weight. Gel B was used for western blotting with a canine serum sample which was seropositive to CNV strain 170 and HK by ELISA.

Western blotting analysis of CNV VLPs using a representative positive canine serum sample was used as an additional method to confirm the specificity of anti-CNV antibodies (figure 5). By ELISA, the selected serum was positive to CNV strains 170 and HK, and this was proven to be the same by western blotting.

### Serological Prevalence by Age

**Figure 6:**
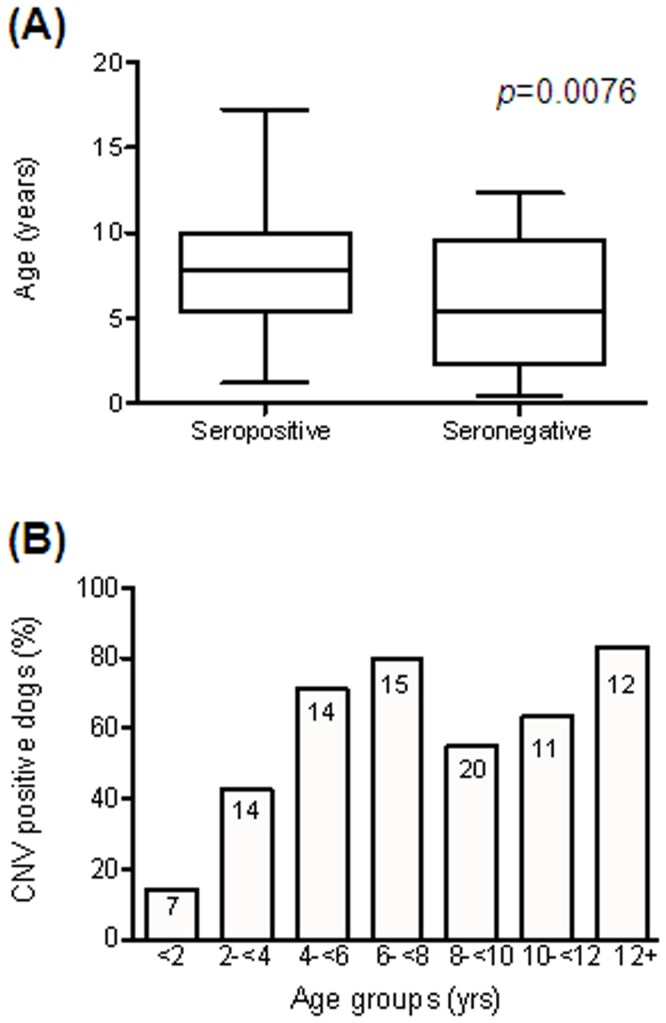
Relationship of CNV antibody status to age. Age was known for 93 dogs in the 2012–2013 cohort. (A) Box plot of age distribution of dogs relative to seroconversion to CNV. The box represents the interquartile range, with the band inside the box representing the median age. The whiskers are the minimum and maximum of all data. (B) Histogram representing the percentage of dogs that have seroconverted to CNV in each age group. Numbers inside bars indicate the quantity of samples associated with each data point.

The age at the time of blood sampling was known for 93 out of the 173 dogs in the 2012–2013 cohort. The mean age of dogs seropositive to CNV was 8.1 years (SD 3.6), whereas the mean age of seronegative dogs was 5.8 years (SD 3.8). The difference between the age distribution of the two groups was statistically significant (*p* = 0.0076, Mann-Whitney test). Division of the dogs into age-groups and calculation of the proportion of each group seropositive to CNV showed that seroprevalence increases from 14% if less than 2 years of age, to almost 80% in the 6–8 year age group (figure 6).

## Discussion

In this study we describe the first evidence that CNV is present in the UK dog population. Whilst previous reports have confirmed the existence of CNV throughout Europe and the US [7], [8], [11], [19], we have now confirmed the presence of CNV-specific antibodies in dogs in the UK, strongly suggesting CNV is circulating.

Seroprevalence to CNV in the canine serum samples collected from 2012–2013 was 60.1%. This is comparable to the seroprevalence to another enteric virus, CECoV, which has been reported to vary between 54% and 78% in a population of UK dogs [20] [21]. CNV seroprevalence was unexpectedly high given that CNV RNA was not detectable in 228 canine stool samples analysed in this study. The high seroprevalence suggests that many dogs are exposed to the virus, with the inability to detect actively secreted viral RNA attributable to two possible factors. Firstly, it is likely that, as with the majority of human norovirus infections in man, CNV infection is very acute with virus only shed during a short period of time. Acute gastroenteritis caused by human norovirus in man results in peak viral shedding at 2–4 days after infection. By 3 weeks after infection only 25% cases are still positive for viral RNA [22]. Preliminary epidemiological data from a study involving a kennel outbreak of CNV in seven dogs found no virus detectable one week after the onset of clinical signs [9]. It is worth noting that although human norovirus is responsible for millions of infections worldwide each year, the virus is only identified in approximately 12% of diarrhoeic samples submitted for analysis [23]. A second explanation for the low prevalence of antigen detection could be due to the genetic heterogeneity of CNV. Significant sequence variation has been shown between the CNV strains characterized so far, and there is evidence of recombination between canine norovirus strains and noroviruses of different genogroups [13]. Design of the primer-probe used in this study was based on a highly conserved region of the CNV genome (NS7), but small sequence differences will affect the ability of the viral sequence to be amplified. Although strains circulating in the UK are antigenically similar to previously identified strains, minor sequence differences could reduce the chance of detection in a PCR screen. To address this, a primer set designed to be broadly reactive to a wide range of noroviruses and sapoviruses (p289/290 [24]) was used to reanalyze the samples. In addition, it must be noted that the primer-probe used in the qPCR survey for CNV RNA was designed prior to the publication of the HK sequence. The HK sequence differences are such that the primer-probe used in the qPCR survey would not detect strain HK. In light of the results of the serosurvey, this issue was addressed retrospectively to the first viral RNA survey. A new primer set was designed to detect the most conserved region of the HK VP1 protein. A SYBR-based qPCR assay was used to screen all 228 stool samples for the presence of any norovirus strain using primer pair p289/290 and also specifically for CNV strain HK using a newly designed primer pair. No positive samples were identified using either primer pair (data not shown).

Seroprevalence to the CNV strains surveyed in this study has been shown to have significantly increased over the past decade in the populations studied. In the 1999–2001 cohort of dogs 38.1% were seropositive, whereas in the 2012–2013 cohort this proportion has almost doubled. Not only does this data provide the first proof that CNV has been present in dogs for at least 8 years prior to its initial discovery [7], it also implies that the number of dogs exposed to these strains of CNV has significantly increased during this period. This conclusion does come with certain caveats however, as the study populations of dogs used are not directly comparable. The 1999–2001 samples were collected from dogs that had been at a rehoming kennels for 3 weeks whereas the 2012–2013 dogs were all privately owned pets. Seroprevalence and overall viral prevalence to many viruses is known to be higher in facilities where dogs are kept in close contact [21] [25]. This does suggest that the apparent increase in seroprevalence to the CNV strains studied is real, as typically owned pets will be exposed less frequently to viruses than dogs in kennels. It would be valuable to assess the current CNV seroprevalence in kenneled dogs, which is predicted to be greater than 60%.

VLPs of three different CNV strains were pooled together to establish the overall seroprevalence to the virus. CNV strains included were identified in 2007 from Italy (170), Portugal (C33) and Hong Kong (HK). Following the initial serosurvey with the pooled VLPs, positive samples were entered into ELISAs with VLPs from each of the individual strains separately. Our data would indicate reactivity to all three strains in the UK dog population. Blocking assays demonstrated no significant cross-reactivity between the three CNV strains in the samples tested, proving that antibodies generated in response to infection were likely to be strain specific. The human immune response against human norovirus is of short duration (up to 14 weeks) and is homotypic, i.e. the immunity acquired for a genogroup or a particular genotype does not provide effective protection against another genogroup or genotype [26]. This study therefore reveals that at least three antigenically distinct CNV strains have been circulating in the UK dog population. This is similar to the co-existence of human norovirus strains in the human population, with both genogroup I and genogroup II strains circulating [27]–[29].

Despite co-existence of multiple human norovirus strains, genogroup II (GII) noroviruses are the most prevalent genogroup worldwide with 96% of human outbreaks attributed to these strains [28]. Our data also identified variation between the prevalence of the different strains, with the highest seroprevalence in both cohorts demonstrated for CNV strain HK (see table 1). This strain has been classified as a GVI norovirus, along with the Portuguese strain C33. However, strain HK has the highest sequence identity (51.6%) to an intergenotype GII recombinant human norovirus strain and it is suggested that strain HK may be classified into a novel genogroup [19]. Aside from this phylogenetic information, it is not possible to determine if the higher seroprevalence to strain HK in our study is due to a viral fitness advantage, and further molecular characterization is required.

The age of seroconversion to CNV shows that exposure to the virus typically occurs in the first few years of life. Seroprevalence increases significantly in older dogs. This is comparable to the seroconversion rates to human norovirus in man; seroprevalence in children less than 2 years old is 20–30%, but this rapidly increases to 70–80% in older children [30], [31]. It is speculated that CNV isolation from stool samples using qPCR will be more likely in a younger cohort of dogs.

To conclude, this report has not only demonstrated that CNV is present within the UK dog population, but has also shown that multiple strains of CNV are co-circulating. Evidence of exposure to CNV prior to its first discovery in 2007 has also been presented, and the rise in seroprevalence over time suggests this pathogen is becoming increasingly common in the UK.
